# Correction to “Improving Sepsis Mortality Prediction With Machine Learning Using Full Region Synthetic Sampling Approach”

**DOI:** 10.1002/hsr2.73075

**Published:** 2026-08-20

I. A. Amory, P. R. Khazaee, and S. Yousefi, “ *Improving Sepsis Mortality Prediction With Machine Learning Using Full Region Synthetic Sampling Approach*,” *Health Science Reports* 8 (2025): 1‐15, https://doi.org/10.1002/hsr2.71556.

The affiliation of the author Saleh Yousefi was incorrectly linked to Affiliation 2. This has been corrected to Affiliation 1.

The online version of this article has been corrected accordingly.

We apologize for this error.

